# Effects of the Food Manufacturing Chain on the Viability and Functionality of *Bifidobacterium animalis* through Simulated Gastrointestinal Conditions

**DOI:** 10.1371/journal.pone.0157958

**Published:** 2016-06-22

**Authors:** Pattra Charnchai, Sirima Suvarnakuta Jantama, Chutinun Prasitpuriprecha, Sunthorn Kanchanatawee, Kaemwich Jantama

**Affiliations:** 1 Metabolic Engineering Research Unit, Institute of Agricultural Technology, School of Biotechnology, Suranaree University of Technology, 111 University Avenue, Suranaree Sub-district, Muang District, Nakhon Ratchasima 30000, Thailand; 2 Division of Biopharmacy, Faculty of Pharmaceutical Sciences, Ubon Ratchathani University, Warinchamrap, Ubon Ratchathani 34190, Thailand; University of Campinas, BRAZIL

## Abstract

The viability and functionality of probiotics may be influenced by industrial production processes resulting in a decrease in probiotic efficiency that benefit the health of humans. This study aimed to investigate the probiotic characteristics of *Bifidobacterium* strains isolated from fecal samples of healthy Thai infants. In the present work, three local strains (BF014, BF052, and BH053) belonging to *Bifidobacterium animalis* showed a great resistance against conditions simulating the gastrointestinal tract. Among these, *B*. *animalis* BF052 possessed considerable probiotic properties, including high acid and bile tolerance, strong adhesion capability to Caco-2 cells, and inhibitory activity against pathogens including *Salmonella typhimurium* and *Vibrio cholerae*. This strain also exhibited a high survival rate compared to commercial strains during storage in a wide variety of products, including pasteurized milk, soy milk, drinking yogurt, and orange juice. The impact of food processing processes as well as the freeze-drying process, storage of freeze-dried powders, and incorporation of freeze-dried cells in food matrix on probiotic properties was also determined. The stability of the probiotic properties of the BF052 strain was not affected by food processing chain, especially its resistance in the simulated gastrointestinal conditions and its adherence ability to Caco-2 cells. It indicates that it satisfies the criteria as a potential probiotic and may be used as an effective probiotic starter in food applications.

## Introduction

The consumption of health-promoting foods has developed in recent years together with an increasing variety of products conferring specific health benefits. In this regard, probiotic-containing foods are highlighted as attractive products due to possess health promotion effects [1,2]. However, before probiotics can be of benefit to the health of humans, they must first be able to survive in sufficient numbers the manufacturing processes and storage as freeze-dried cultures, and also in the food products into which they are finally formulated. In addition, they should also possess the ability to survive the gastrointestinal (GI) tract and retain their functionality to be effective in the host [3,4]. Consequently, probiotic strains selected for commercial applications must retain the characteristics for which they were originally selected [5]. Recently, du Toit et al. [6] demonstrated that the same probiotic strain presented different characteristics depending on the manufacturing and processing conditions.

The manufacture of bifidobacterial cells at the recommended level of 10^6^–10^7^ CFU/ml of product represents a major technological challenge due to several factors that affect the viability of bifidobacteria during manufacturing and storage, such as the presence of oxygen, temperature, pH, and osmotic changes [2,7]. Production technology, in particular freeze-drying, manufactures powders containing a high number of viable cell cultures. Moreover, bifidobacterial cultures need to be able to survive during the shelf-life of the products [8]. The viability and stability of probiotics are challenges for industrial producers, and new technology has been developed to obtain highly stable probiotic starters with stable functionality [2,3,9]. A rigorous effort in strain selection and characterization is regarded as a prerequisite in this process [10]. This reinforces the need for robust bifidobacteria that are able to survive stressful environmental challenges not only during industrial processes such as freeze-drying, manufacturing, and storage but also after consumption through the GI tract stresses, until their adherence to the intestinal epithelium to exert health-promoting effects there [4]. Therefore, to guarantee a functional and effective probiotic strain with predictable health benefits, its viability and functionality throughout the food manufacturing processes and GI stress barriers must be investigated to ensure that health-promoting properties are maintained.

The aim of the present work was to investigate the probiotic characteristics of *Bifidobacterium* spp. previously isolated from fecal samples of healthy Thai infants as a highly stable probiotic starter. The candidate strains were initially screened on the basis of acid and simulated gastric tolerance and were further screened for functional properties, such as antimicrobial activity and adhesion ability. In addition, the study investigated the viability of the probiotic strains during storage in different food matrices. The authors consider this study to be a pioneer work in the evaluation of the impact of the production process chain on the selected probiotic’s survival and resistance to GI stress, and its adhesion ability to Caco-2 cells.This part of the study was to ensure that the strain would still provide probiotic effects after consumption.

## Materials and Methods

### Microorganisms and culture conditions

Thirty bifidobacterial strains identified as *Bifidobacterium animalis* were selected from Suranaree University of Technology (SUT) culture collection, Thailand. These strains were previously isolated from fecal samples of healthy Thai infants. The widely used *Bifidobacterium animalis* subsp. lactis, strain BB-12 (Bb12) (Chr. Hansen, Denmark) was included in the experiments for comparison purposes. All strains were grown anaerobically at 37°C in DeMan, Rogasa and Sharpe (MRS; Oxoid Ltd., UK) broth supplemented with 0.05% L-cysteine hydrochloride (MRSc) and maintained in MRSc broth containing 20% (v/v) sterile glycerol and stored at -80°C.

The indicator organisms used for antimicrobial activity included *Escherichai coli* TISTR 780, *Staphylococcus aureus* TISTR 1466, *Pseudomonas aeruginosa* TISTR 781, *Bacillus cereus* TISTR 687, *Samonella typhimurium* TISTR 292, *Vibrio cholerae* O139, and *Candida albicans* TISTR 718 which were supplied from the culture collection of the Laboratory of Microbiology, Institute of Science, Suranaree University of Technology, Thailand. All strains were cultured on Brain-Heart Infusion agar (BHI; Conda-Pronadisa, Spain) at 37°C for 16 h.

### Resistance under conditions simulating the human gastrointestinal tract

The resistance of the examined strains under conditions simulating the GI tract was tested as previously described [11]. The tolerance was initially screened through low pH and simulated gastric juice. Briefly, bacterial cells from overnight (18 h) cultures were harvested (4,000 rpm, 10 min, 4°C) and washed twice with phosphate buffered saline (PBS; 0.8% NaCl, 0.2% KCl, 0.144% Na_2_HPO_4_, 0.024% KH_2_PO_4_, pH 7.2) supplemented with 0.05% L-cysteine hydrochloride (Merck, Germany) (PBSc), before being re-suspended in PBSc solution and adjusted to pH solutions of 2 and 3. For resistance to simulated gastric juice, bacterial cells were harvested and washed as described above. The bacterial suspension was then re-suspended in PBSc solution containing 0.3% (w/v) pepsin (Sigma-Aldrich, USA) and adjusted to pH solutions of 2 and 3. Resistance was assessed in terms of viable colony counts on MRSc agar after incubation of bacterial suspensions at 37°C for 0 and 3 h, reflecting the time spent by food in the stomach.

For resistance to small intestine conditions, bacterial cells as prepared above were re-suspended in PBSc solution containing 0.1% (w/v) pancreatin (Sigma-Aldrich, USA) and pH 8. The ability of the isolates to grow in the presence of bile was determined by adding cell suspensions to MRSc broth supplemented with 0.3%, 0.5 and 1% (w/v) bile salt (Oxoid Ltd., UK) and pH 8. The viable colony counts were determined after incubation at 37°C for 0 and 4 h, reflecting the time spent by food in the small intestine.

### *In vitro* adherence assay

An adherence ability of the bifidobacteria was examined *in vitro* using Caco-2, a colonic adenocarcinoma cell line that expresses the morphological and physiological characteristics of normal mature human enterocytes. An adhesion assay was conducted as previously indicated by Pennacchia et al. [12]. Caco-2 cells were routinely grown in Dulbecco’s modified Eagle’s minimal essential medium (DMEM; Gibco, USA) supplemented with 10% (v/v) heat inactivated fetal bovine serum, 1% (v/v) L-glutamine, 1% (v/v) non-essential amino acid solution, and 1% (v/v) penicillin/streptomycin solutions (Gibco, USA) at 37°C in 5% CO_2_ and 95% air atmosphere. Before the adhesion assay, overnight cultures of bacterial strains were harvested by centrifugation at 4,000 rpm and 4°C for 10 min (Centrifuge 5810R, Eppendorf, Germany). An aliquot of culture suspensions was serially diluted 10-fold in PBSc to determine the viable population by plate counting on MRSc agar after 48 h of incubation at 37°C. Another aliquot was re-suspended in non-supplemented DMEM (pH 7.0). This bacterial suspension was used to inoculate the six-well tissue culture plates with a concentration of about 10^8^ CFU/mL.

The monolayer Caco-2 cells in the six-well tissue culture plates were washed twice with PBS and 2 ml of non-supplemented DMEM was added to each well. The plate was incubated at 37°C for 1 h. After incubation, non-supplemented DMEM was removed from each well and replaced by 1 ml of the bacterial suspension, prepared as described above. After incubation at 37°C for 90 min, the wells were softly washed 3 times with PBS to remove non-adherent bacteria. The washed monolayer was treated with 1 ml of 0.05% Triton X-100 water solution for 10 min to lyse the Caco-2 cells. The number of viable adhering bacteria was determined by plating serial 10-fold dilutions of the mixture containing lysed Caco-2 cells and bacterial cells on MRSc agar after 48 h of incubation at 37°C. The adhesion ability of the strains on Caco-2 cells was calculated as a percentage of the viable bacteria according to their initial population.

### Antibiotic susceptibility test

Antibiotic susceptibility patterns of the strains were investigated by the disk diffusion method. The tested antibiotic discs (Oxoid, England) included streptomycin (10μg), gentamicin (10μg), tetracycline (30μg), penicillin G (10μg), aztreonam (30μg), vancomycin (30μg), erythromycin (15μg), chloramphinicol (30μg), kanamycin (30μg), ampicilin (10μg), lincomycin (15μg), norfloxacin (10μg), and ofloxacin (5μg). Strains were grown in MRSc broth at 37°C for 24 h under anaerobic condition to obtain a density of 10^7^ cfu/mL. The culture suspension was swabbed on MRSc agar. Antibiotic discs were placed aseptically on the inoculated plates and agar plates were incubated anaerobically for 24 h at 37°C. The diameters of the inhibition zones around the discs were measured (average of three readings) and the results were interpreted according to the Clinical and Laboratory Standards Institute (CLSI, 2014) as sensitive (S), intermediate (I), and resistant (R).

### Antimicrobial Activity

The ability of the candidate strains to inhibit the growth of pathogenic microorganisms was determined using the agar-well diffusion assay [13]. An overnight culture of the indicator strains was applied to inoculate in BHI agar at 37°C. Fresh overnight bifidobacteria cultures were harvested by centrifugation (4,000 rpm, 10 min, 4°C). The supernatants were neutralized to pH 6.5 and the others left unadjusted followed by filter-sterilization through 0.22 μm membrane filter. Cell-free extracts of bifidobacteria samples (100 μL) were pipetted into drilled holes (7mm) of the agar. The plates were then incubated at 37°C and were examined after overnight incubation. Antimicrobial activity was recorded as growth-free inhibition zones (mm) around the well.

### Stability of probiotics in commercial products during storage

All candidate bifidobacteria were propagated in MRSc broth overnight at 37°C followed by sub-culturing and incubating for a further 18 h. All cultures were harvested by centrifugation and the pellets were then washed twice in PBSc solution, pH 7.4. A 1% inoculum of each bifidobacterial culture was aseptically distributed into 100 mL portions of four commercial dairy and non-dairy products (pasteurized milk, drinking yogurt, soy milk, and orange juice) to obtain a final concentration of 10^7^−10^8^ CFU/mL. Cell counts and pH measurements were performed immediately after the addition and every three days until 15 days of storage at a refrigerated temperature.

### Preservation of bifidobacteria by freeze-drying in different cryoprotectants

A fresh overnight culture of a selected probiotic strain, *B*. *animalis* BF052, was grown in MRSc broth at 37°C. A 1% inoculum was then subsequently transferred to fresh MRSc broth. At the early stationary phase of growth (18 h), cells were harvested by centrifugation and washed twice with PBSc solution, pH 7.4. The pellet was re-suspended in 10% (w/v) lactose, 10% (w/v) sucrose, 10% (w/v) skim milk, 10% (w/v) germinated brown rice (GBR), 10% (w/v) black sesame (BS), and commercial soy milk. Sterile de-ionized water was used as a control. Aliquots (1 ml) of each cell suspension in different cryoprotectants were transferred into sterilized vials and frozen at -80°C for 4 h. Then, the samples were immediately freeze-dried for 18 h in a freeze-dryer (Alpha 1–2, Christ, Germany).

After freeze-drying, the freeze-dried powders were re-hydrated with MRSc broth (1 ml) and the cell suspensions were allowed to stand for 10 min at room temperature, and subsequently plated on MRSc agar. The number of viable cells before and after freeze-drying was determined at 37°C after incubation for 48 h. To select the most effective cryoprotectant, freeze-dried samples were kept at room and refrigerated temperatures. After storage for 1, 3, and 6 months, the viability of the freeze-dried cells was then determined by plating on MRSc agar after 48 h of incubation at 37°C.

### Gastrointestinal transit tolerance of BF052

The study investigated the effects of the production process chain, freeze-drying, storage of freeze-dried powders, and incorporation of cells in food matrix on the stability of the probiotic properties of *B*. *animalis* BF052. The strain *B*. *animalis* BF052 was subjected to a process of freeze-drying. The skim milk (10%) was used as a cryoprotective agent and the freeze-dried powders were then stored for 1 month following incorporation into a whole pasteurized milk and kept at refrigerated temperatures for 2 weeks. The strains were then sequentially exposed to simulated GI conditions followed by the adherence assay. To mimic *in vivo* human GI transit, an *in vitro* model was conducted as previously described by Peres et al. [1] and Sousa et al. [2], with slight modifications. After incorporation of the freeze-dried cells in a whole pasteurized milk for 2 weeks, 1 ml of products were transferred to a 34 ml of sterile electrolyte solution (SES; 0.22 g L^-1^ CaCl_2_, 6.2 g L^-1^ NaCl, 2.2 g L^-1^ KCl, 1.2 g L^-1^ NaHCO_3,_ w/v) adjusted to pH 6.2. To simulate *in vivo* saliva conditions, 5 mL of a sterile electrolyte solution containing lysozyme (final concentration of 0.01% w/v) was added to 35 mL of cell suspension and incubated at 37°C, 200 rpm for 2 min. Then, 3 mL of the electrolyte solution (pH 5.0) with 0.3% (w/v) pepsin was incorporated into the cell suspension to simulate the oesophagus-stomach environment. The pH curve in the stomach was reproduced by adding 1 N HCl to the cell suspension to pH 6.0, 5.0, 4.0 every 10 min and to pH 3.0, 2.0 every 30 min, at 37°C, 50 rpm respectively. After 90 min of incubation, the samples were then adjusted to pH 5.0 using 1 M NaHCO_3_ and mixed with 4 mL of a sterile electrolyte solution (5 g/L NaCl, 0.6 g/L KCl and 0.3 g/L CaCl_2,_ w/v), containing 0.3% (w/v) bile salts and 0.1% (w/v) pancreatin (pH 8) and incubated for 30 min (37°C and 50 rpm) to simulate the intestinal environment at the duodenum step. Finally, the ileum step was brought about by an increase of pH to 6.5 and incubation for 90 min at 37°C and 50 rpm_._ After passing through the GI step, cells were then sequentially tested for adherence ability. Briefly, bacterial solution was centrifuged and the pellet was re-suspended in 2 ml of non-supplemented DMEM. The monolayer Caco-2 cells in the six-well tissue culture plates were washed twice with PBS and 2 ml of non-supplemented DMEM was added to each well. The plate was incubated at 37°C for 1 h. After incubation, the non-supplemented DMEM was removed from each well and replaced by 2 ml of the bacterial suspension. After incubation at 37°C for 90 min, the wells were washed 3 times with PBS to remove non-adherent bacteria. The washed monolayer was treated with 2 ml of 0.05% Triton X-100 water solution for 10 min to lyse the Caco-2 cells. The number of viable adhering bacteria was enumerated by plating on MRSc agar after 48 h of incubation at 37°C.

### Statistical analysis

Data were analyzed using SPSS 16.0 software (SPSS Inc., Chicago, IL, USA). Statistical differences in multiple groups were determined by one-way ANOVA followed by multiple mean comparisons with Duncan’s test. All numerical data were displayed as mean ± standard deviation and p ≤ 0.05 was considered statistically significant.

## Results and Discussion

### Resistance of bifidobacteria under conditions simulating the gastrointestinal tract

An essential step towards the selection of potential probiotic candidates is to examine their resistance under GI stress environments [1,14]. Out of 30 strains, only BF014, BF049, BF052, and BH053, including the reference strain Bb12, showed a decrease in viable counts lower than 1 log cycle even after 3 h of exposure at pH 3 (Table 1). No significant differences (p>0.05) in viable cells were observed in BF052 and Bb12 after an exposition to solutions with or without pepsin at pH 3 compared with initial counts. Although BF014, BF052, and BH053 did not survive after exposure at pH 2 for 3 h, all of the three strains consistently tolerated the pepsin solutions at pH 2 after 3 h of incubation. These results indicated that the bifidobacterial isolates (except strain BF049) were able to tolerate simulated gastric juice at pH 2 in the presence of pepsin. This result is relevant to the work of Mättö et al. [15] that showed the addition of inhibitors of pepsin and proton translocating enzyme significantly decreased the survival rate of *B*. *animalis* subsp. *lactis* at pH 2. Therefore, it was likely that pepsin was able to protect the cells during exposure to low pH by maintenance of the pH homeostatis and support of the role of H^+^-ATPase. However, the loss of viability in BF049 after exposure to simulated gastric juice may indicate that the resistance to enzymatic barriers was strain-specific.

**Table 1:** Cell viability of probiotic strains after 3 h of exposure to low pH conditions and simulated gastric juice.

Strain	Initial count	low pH conditions		Resistance to gastric juice with 0.3% (w/v) pepsin	
		pH 2	pH 3	pH 2	pH 3
Bb12	6.71 ± 0.02^φ^^,a^	-^γ^	6.61 ± 0.05^a^	6.21 ± 0.04^b^	6.65 ± 0.01^a^
BF014	7.14 ± 0.11^a^	-	6.86 ± 0.25^ab^	6.46 ± 0.05^b^	7.08 ± 0.12^a^
BF049	7.13 ± 0.03^a^	-	6.93 ± 0.02^b^	-	1.61 ± 0.02^c^
BF052	7.31 ± 0.06^a^	-	7.25 ± 0.07^a^	6.99 ± 0.02^b^	7.26 ± 0.05^a^
BH053	7.79 ± 0.06^a^	-	7.34 ± 0.18^b^	7.19 ± 0.03^b^	7.65 ± 0.03^a^

^φ^ Each value represents the mean value (log CFU/mL) ± stand deviation (SD) from three trials. The equal superscript lowercase letters in the same row indicate no significant differences (p>0.05)

^γ^ No growth.

The strains resistant to stomach conditions were further tested for their ability to tolerate small intestinal conditions. All of the candidate strains were resistant to bile salts and pancreatic solutions at pH 8 by decreasing their viability approximately 1 log unit after 4 h exposure, as shown in Table 2. In general, the relevant physiological concentrations of human bile range from 0.3% to 0.5%. However, it was reported that bile salts were critical to bacterial cells since they disorganized the structure of the cell membrane [16]. However, all tested strains in this study retained their viability with small reductions at high concentrations of bile ranges from 0.3% to 1%.

**Table 2:** Cell viability of probiotic strains after 4 h of exposure to bile salt and pancreatin.

Strain	Initial count	Bile salt (pH 8)			Pancreatin (pH 8)
		0.3%	0.5%	1%	
Bb12	7.42 ± 0.14^φ^^,a^	6.45± 0.08^b^	6.43 ± 0.04^b^	6.41 ± 0.09^b^	6.42 ± 0.27^b^
BF014	7.28 ± 0.12^a^	6.51 ± 0.26 ^b^	6.38 ± 0.03 ^b^	6.43 ± 0.02 ^b^	6.15 ± 0.14 ^b^
BF049	7.27 ± 0.08^a^	6.58 ± 0.03 ^b^	6.58 ± 0.07 ^b^	6.32 ± 0.09^c^	5.10 ± 0.11^d^
BF052	7.44± 0.06 ^a^	6.80 ± 0.02 ^b^	6.77 ± 0.01^b^	6.73 ± 0.03 ^b^	6.32 ± 0.15 ^c^
BH053	7.43 ± 0.10^a^	6.25 ± 0.18 ^b^	6.23 ± 0.12^b^	6.23 ± 0.15 ^b^	6.31 ± 0.15 ^b^

^φ^ Each value represents the mean value (log CFU/mL) ± stand deviation (SD) from three trials. The equal superscript lowercase letters in the same row indicate no significant differences (p>0.05).

In this study, three strains, BF014, BF052, and BH053, showed satisfactory probiotic properties for preliminary screening under conditions simulating the GI tract, suggesting that they may survive through the human GI transit. All of these three strains were therefore selected for the study of other probiotic properties.

### Caco-2 cell adhesion

Adhesion of probiotic strains to human intestinal mucosa is regarded as a prerequisite characteristic for potential probiotic microorganisms. The adhesion ability to Caco-2 cells was evaluated and the result is presented in Table 3. The BF052 strain had a significantly higher adherence (3.38% ± 0.15) to Caco-2 cells comparable with the reference strain Bb12 (2.96% ± 0.12), whereas BF014 and BH053 expressed lower levels of adhesive abilities than those of BF052 and Bb12 strains. Sánchez et al. [17] revealed that adhesion values to the intestinal cell line HT29-MTX by *B*. *animalis* subsp. lactis IPLA4549 (2.96%±1.74) was slightly lower than Bb12 (3.08%±1.37). In addition, Laparra and Sanz [18] reported that Bb12 showed the highest adherence capability to Caco-2 cell and to human mucus (mucin type II) compared with other probiotic strains including *Lactobacillus rhamnosus* GG, *B*. *animalis* IATA-A2 and *B*. *bifidum* IATA-ES2. In this study, BF052 showed the highest percentage of adhesion compared to those candidate strains including the reference strain. As previously reported, the adhesion capability was not associated with species but as a characteristic of strain [19].

**Table 3:** Adhesion ability of the isolates to Caco-2 cells.

Strain	% adhesion (mean±SD)*
Bb12	2.96 ± 0.12^a^
BF014	2.57 ± 0.38^b^
BF052	3.38 ± 0.15^c^
BH053	2.72± 0.37^ab^

*The equal superscript lowercase letters in the column indicate no significant differences between strains (p>0.05).

The adhesion of the microorganisms to the intestinal mucosa is an important feature involved in colonization and is related to the ability of the strains to interact with the host [20]. Probiotic bifidobacteria have several mechanisms that enable them to adhere to the intestinal epithelial cells. Their possible mechanisms may confer competition for substrates, direct antagonism by inhibitory substances, competitive exclusion of pathogenic bacteria, and potentially host-mediated effects, such as enhancing the function of the intestinal epithelial barrier by stimulation of the various signaling pathways and modulating immune responses [21–23]. As a result, high adhesive ability of bacteria to the cell lines may indicate that strains may contribute their beneficial effects to the host. However, investigations are still necessary to confirm their functionality in *in vivo* situations.

### Antibiotic susceptibility assay

An important requirement for probiotic strains is that the isolated probiotics must be safe for human consumption. In this regard, antibiotic susceptibility profiles should be revealed and taken into account for safety [24]. Table 4 lists the antibiotic susceptibility patterns of the candidate strains and all candidates displayed similar phenotypic resistances comparable with the reference strain, Bb12. All tested strains were interpreted to be resistant towards aminoglycoside group (streptomycin, gentamycin, kanamycin), fluoroquinolone antibiotics (norfloxacin and ofloxacin), and β-lactam antibiotic (aztreonam, which is gram-negative spectrum). In contrast, all strains were sensitive to antibiotics belonging to a broad range of antibiotics related to different modes of action, such as β-lactam antibiotics (penicillin and ampicilin), broad-spectrum antibiotics (tetracycline and chloramphenicol), macrolide antibiotic (erythromycin), glycopeptide antibiotic (vancomycin), and lincosamide antibiotic (lincomycin). These antibiotic results indicated related patterns to previous reports [5,25,26]. From a safety point of view, it was proposed that a prospective probiotic should not carry transmissible antibiotic resistance genes, resulting in the corresponding genes not being transferred to the others including pathogens and commensal gut microbiota [27]. Probiotic strains with intrinsic antibiotic resistance may be thus useful for the restoration of the gut microbiota after antibiotic treatment [5]. Moreover, to the best of our knowledge, this is the first report in which all tested strains conferred resistance to norfloxacin and ofloxacin. Therefore, it is beneficial for patients suffering from urinary tract infection to restore the *Bifidobacterium* population after treatments involving norfloxacin and ofloxacin.

**Table 4:** Antibiotic susceptibility profiles.

Type of antibiotics	Antibiotic susceptibility profiles			
	Bb12	BF014	BF052	BH053
Streptomycin (10μg)	R	R	R	R
Gentamicin (10μg)	R	R	R	R
Tetracycline (30μg)	S	S	S	S
Penicillin G (10μg)	S	S	S	S
Aztreonam (30μg)	R	R	R	R
Vancomycin (30μg)	S	S	S	S
Erythromycin (15μg)	S	S	S	S
Chloramphinicol (30μg)	S	S	S	S
Kanamycin (30μg)	R	R	R	R
Ampicilin (10μg)	S	S	S	S
Lincomycin (15μg)	S	S	S	S
Norfloxacin (10μg)	R	R	R	R
Ofloxacin (5μg)	R	R	R	R

### Antimicrobial Activity

For the antimicrobial assay, there was no observation of inhibition for any of the supernatants in which the pH was neutralized (results not shown). However, the non-neutralized culture supernatants of BF052 and BH053 strains showed inhibitory activities against *S*. *typhimurium* and *V*. *cholerae* as shown in Table 5. These results indicated that the most likely explanation was that the inhibition was due to organic acid production by the strains. Our results were in an agreement with previous works. Strompfová and Lauková [28] demonstrated that inhibition effects were not explained by bacteriocin action and were most probably due to the production of organic acids along with pH lowering effects during the growth in *Bifidobacterium*. Arboleya et al. [25] also reported that non-neutralized supernatants of breast-milk isolates (*B*. *longum* and *B*. *breve*) were able to inhibit *Salmonella enteric* and *Shigella sonnei*. Ibrahim and Bezkorovainy [29] demonstrated that no antibacterial substances were detected in the fermentation broth of tested bifidobacteria. Only acetic and lactic acids were produced and could inhibit the pathogenic strain of *E*. *coli*. In addition, Fukuda et al. [30] proposed that the production of acetate by *B*. *longum* subsp. longum JCM 1217, *B*. *longum* subsp. infantis 157F, and *B*. *longum* subsp. longum NCC 2705 was able to protect mice against death induced by enterohaemorrhagic *Escherichia coli* O157:H7. However, Liu et al. [31] recently found a novel broad-spectrum bacteriocin called bifidocin A that is produced by *B*. *animalis* BB04. Therefore, it is likely that the antimicrobial activity of bifidobacteria may be implemented not only by the production of organic acids but also by the secretion of bacteriocin.

**Table 5:** Inhibitory effects of non-neutralized bifidobacterial supernatants against pathogens.

Strains	Diameter (mm) of inhibition zones						
	*E*. *coli*	*S*.*aureus*	*P*. *aeruginosa*	*V*. *cholerae*	*B*. *cereus*	*S*. *typhimurium*	*C*. *albicans*
Bb12	-^a^	-	-	-	-	-	-
BF014	-	-	-	-	-	10	-
BF052	-	-	-	8	-	10	-
BH053	-	-	-	9	-	11	-

^a^ No antagonistic activity was observed.

### Storage stability of probiotics in commercial products

Many criteria have been suggested for the selection of probiotics. Besides the challenge to overcome the GI stresses, the ability of probiotics to survive in products during storage is also important. It was recommended that the level of probiotics in food products needed to be high, suggesting the minimum counts of live cells should be at least 10^6^−10^7^ CFU/mL before consumption [2, 32]. This requirement has a significant impact on the selection of potential probiotics with high stability in different food products.

**Fig 1:**
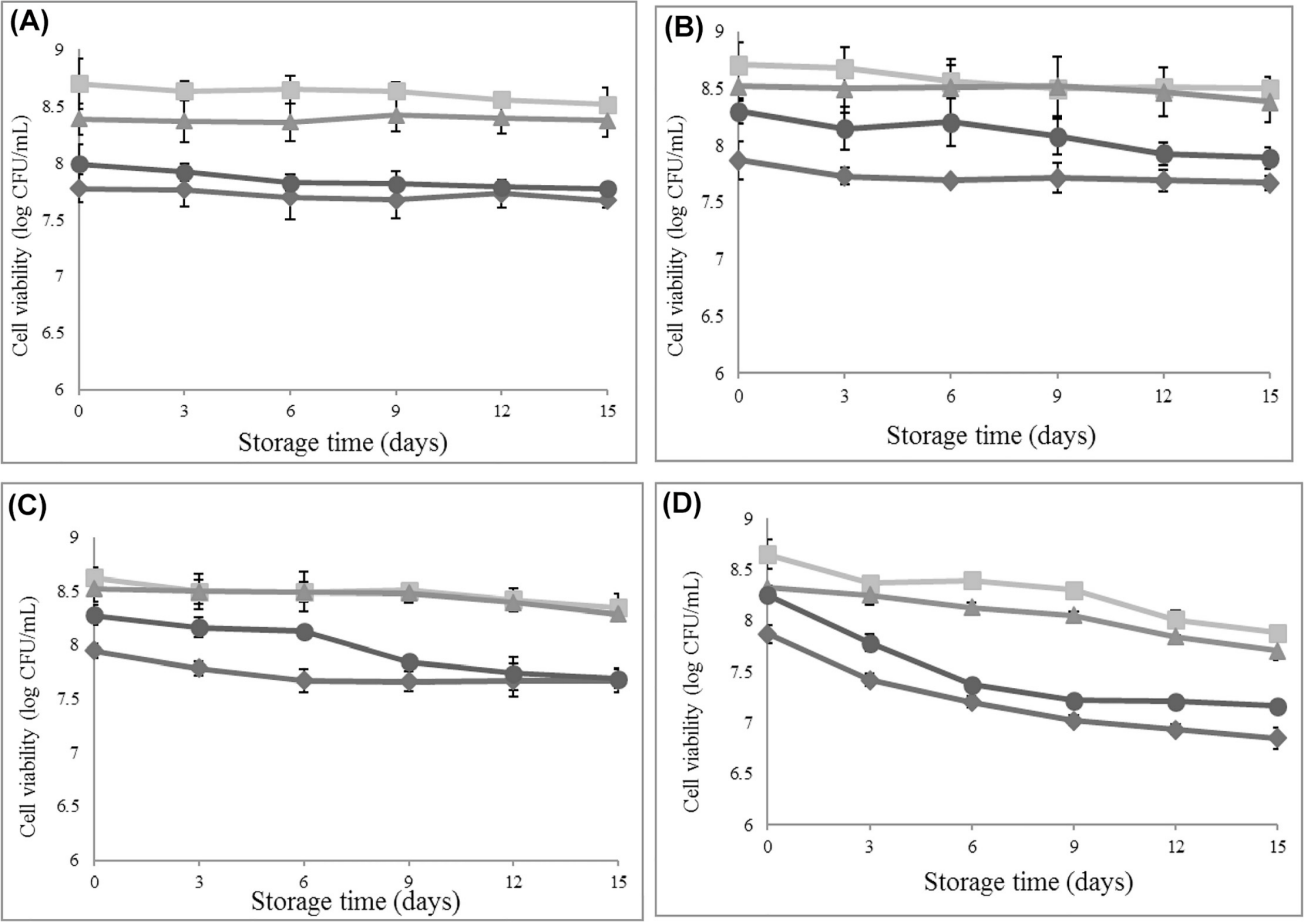
**Cell viability of probiotics in refrigerated storage over 15 days in (a) pasteurized milk, (b) soy milk, (c) drinking yogurt and (d) orange juice**. Symbols: Bb12 (◆), BF014 (■), BF052 (▲), and BF053 (●).

In the present study, strains Bb12, BF014, BF052, and BH053 were incorporated into dairy (pasteurized milk and drinking yogurt) and non-dairy products (soy milk and orange juice) at refrigerated temperatures for 15 days. Fig 1 displays viable cells in refrigerated storage over 15 days. No significant differences (p>0.05) were observed in all the candidate strains in cultivable cell numbers during storage in pasteurized milk and soy milk during the 15 days. In drinking yogurt, a significant decrease (p≤0.05) in cell viability was detected only in strain BH053 after storage for nine days (S1 Table). A major significant reduction (p≤0.05) in cell counts ranging from 0.6 to 1.0 log cycles was observed in orange juice in all tested strains. These results are in agreement with those of Saarela et al. [33] and Vinderola et al. [34] who reported that the stability of bifidobacterial cells in the low pH of fruit juice was poorer than the fairly neutral pH of milk during refrigerated storage. Nualkaekul et al. [35] proposed that the presence of protein sources in food matrices may improve the survival of bifidobacteria during refrigerated storage. This was in accordance with the present study’s findings that high amounts of proteins in drinking yogurt may have resulted in a higher rate of cell survival than in juices, although pH values of both products were slightly different. It was proposed that when probiotic cells were present in low pH environments, the requirement of energy consumption increased to maintain the intracellular pH, resulting in depression of ATP for crucial cellular functions and thereby causing cell death. In addition, exposure to oxygen under acidic conditions during refrigeration storage was most probably responsible for the reduction in probiotic counts [36, 37]. Among all the candidates, BF052 showed the highest survival rate during storage in all products, while the reduction rates of BH053 in terms of viable counts were significantly higher than those of other strains.

Additionally, changes of pH values of BH053 slightly declined compared to those of other strains, especially in soy milk, whereas BF052 remained constant during the incubation period (data not shown). The reduction of the pH value of BF052 in only soy milk (within 0.17 pH-values) but no other products was observed. This result was in line with previous studies which showed the decrease of the pH levels in the soy beverage was faster than in milk [38, 39] suggesting a greater rate of organic acid production. It was also observed that soy milk containing oligosaccharides, such as raffinose and stachyose, may support the growth of bifidobacteria causing acid production and subsequent reduction of pH. However, post-acidification during storage is an undesirable property in probiotic-containing products. This process may have adverse effects on the taste or aroma of the product and may cause a loss in the viability of the probiotic strain [40].

The main purpose of the present study was to identify suitable probiotic strains for incorporation into food products. It was clearly observed that candidate strains belonging to the same species may present different characteristics even in food matrices. Among all candidates, BF052 was found to exhibit the highest survivability in a wide variety of the products, suggesting that it may have been present in sufficient amounts throughout the entire shelf life of the product. In addition, BF052 possessed considerable probiotic properties including high acid and bile tolerance ability, strong adhesion capability, and good inhibitory activity against pathogens. This strain was thus selected as a promising probiotic strain that may have potential as probiotic starter.

### Preservation of BF052 by freeze-drying in different cryoprotectants

In industrial applications, the use of probiotics as starter cultures is required to guarantee long-term delivery of stable cultures in terms of cell viability and functionality [41]. Freeze-drying is a well-documented technique used for the preservation of microorganism [42]. Moreover, the utilization of a suitable cryoprotective as a freeze-drying agent is an achievable attempt to improve cell viability during this process. In this study, sucrose, lactose, skim milk, GBR, BS, and soy milk were examined for their ability to protect the BF052 cells during freeze-drying. Table 6 shows the effect of cryoprotectants on the survivability of BF052 at different storage periods and temperatures.

**Table 6:** Effects of cryoprotective agents on cell survival of BF052 during freeze-drying (FD) and storage.

Cryoprotectants	Cell viability (log CFU/mL ± SD)		Cell viability after storage in refrigerator (log CFU/mL ± SD)			Cell viability after storage at room temperature (log CFU/mL ± SD)		
	Before FD	After FD	1 month	3 months	6 months	1 month	3 months	6 months
DI water	9.32 ± 0.07^a^	8.60 ± 0.2-^b^	8.42 ± 0.02^b^	8.10 ± 0.10^c^	7.79 ± 0.04^d^	6.09 ± 0.08^e^	-	-
10% Sucrose	9.32 ± 0.27^a^	9.02 ± 0.01^b^	8.73 ± 0.01^c^	8.68 ± 0.01^cd^	8.44 ± 0.02^d^	6.26 ± 0.19^e^	-^π^	-
10% Lactose	9.34 ± 0.22^a^	8.99 ± 0.10^b^	8.75 ± 0.02^bc^	8.76 ± 0.08^bc^	8.65 ± 0.04^d^	7.80 ± 0.03^e^	6.02 ± 0.16^f^	3.10 ± 0.04^g^
10% Skim milk	9.21 ± 0.07^a^	9.16 ± 0.02^a^	9.16 ± 0.01^a^	9.15 ± 0.01^a^	9.12 ± 0.01^a^	8.78 ± 0.10^b^	7.42 ± 0.08^c^	6.06 ± 0.03^d^
10% Germinated brown rice	9.81 ± 0.32^a^	9.41 ± 0.23^ab^	9.19 ± 0.12^bc^	8.90 ± 0.10^c^	nd	7.61 ± 0.19^d^	5.14 ± 0.13^e^	nd
10% Black sesame	9.36 ± 0.03^a^	8.48 ± 0.65^b^	8.39 ± 0.06^b^	8.31 ± 0.08^b^	nd	7.76 ± 0.16^b^	6.58 ± 0.15^c^	nd
soy milk	9.20 ± 0.27^a^	8.93 ± 0.10^ab^	8.87 ± 0.09^ab^	8.83 ± 0.09^ab^	nd	8.77 ± 0.07^ab^	7.33 ± 0.14^c^	nd

- The equal superscript lowercase letters indicate no significant differences between cryoprotectant (p>0.05)

^π^ No growth

^nd^ Not determined.

Among all candidate cryoprotectants, only 10% skim milk showed no statistical difference (p>0.05) in protecting cells during freeze-drying. After storage, the survival rates of the BF052 freeze-dried cells were better at refrigerated temperatures than room temperature. It was reported that powdered *Bifidobacterium* preparations survived better in refrigerated storage than at room temperature [33]. In addition, there were no significant differences (p<0.05) in the viable cells using soy milk and BS after storage of freeze-dried powders for 1 month at refrigerated temperature and room temperature. However, after a month storage, the cell viability after 1 month storage in soy milk was higher than that of BS.

According to Carvalho et al. [43], distinct properties of the cryoprotectants resulted in different protection features. The protective ability of skim milk on freeze-dried cells may be explained by its capacity in the prevention of cellular injury, stabilization of the cell membrane constituents, and provision of a protective coating for the cells [44]. Vinderola et al. [33] also found that skim milk and lactose were effective in the protection of *B*. *animalis* subsp. lactis INL1 comparable with sucrose during freeze-drying and storage, including after exposure under the harsh conditions of simulated digestion. Besides skim milk, soy milk is especially interesting as an attractive cryoprotectant. It is likely that soy milk contains many substances in protecting BF052 freeze-dried cells, such as protein, which is equivalent to that of milk, soybean-oligosaccharides, stachyose, and raffinose. Moreover, it was reported that the survival of *B*. *animalis* subsp. lactis 10140 during the freeze-drying process was enhanced by the presence and increment of probiotics [45]. In contrast to skim milk and soy milk, GBR and BS are composed of mostly polymeric sugars. They easily form glasses that often do not have suitable structures to be able to depress membrane phase transition resulting in failure to protect microbial cells during the freeze-drying process [46]. Zhao and Zhang [41] suggested that a good cryoprotection should protect the cells during the freezing process, be easily dried, and provide a good matrix to allow stability and ease of rehydration. During rehydration using MRS broth, GBR and BS were not perfectly rehydrated due to complex substances and thus affected the survival rate of freeze-dried cells.

Nowadays, the demand for non-dairy probiotic products has increased and the use of soy milk as a cryoprotectant during the freeze-dried process is an option to develop a fully non-dairy probiotic product. However, dairy products are still the main vehicles for the incorporation of probiotic cultures [5, 47]. In this study, skim milk was the most effective protective agent for BF052 cells during freeze-drying and storage, and was therefore selected for further study.

### Gastrointestinal transit tolerance of BF052

This study aimed to examine the consistency of the probiotic properties of BF052 after the production process, including freeze-drying, storage, and incorporation of the strain into the products. After this process, BF052 was evaluated the tolerance ability through an *in vitro* model of the human GI tract. The strain was encountered the lysozyme-containing saliva in the mouth, pH gradient and gastric enzymes in the stomach, followed by the bile and pancreatic enzymes in the small intestine, and the adherence of the strain to human intestinal mucosa as a final step. Changes in cell viability by the end of each stage were examined.

Strain BF052 showed the ability to resist to the adverse conditions tested in every compartment as shown in Table 7. It exhibited a small susceptibility through each step, with different enzymatic- and pH-dependent barriers until gastric emptying at increasingly lower pH (reaching to pH 2.0). A significant reduction (p≤0.05) in cell survival occurred only at pH 2 in all processes. This strain was also resistant to the duodenum and ileum steps and retained its viability with a small reduction in viable counts. These results are consistent with those previously revealed from other *B*. *animalis* strains belonging to *B*. *animalis* Bb12 [2] and *B*. *animalis* Bo [48], which generally showed a great resistance throughout the whole processes of simulated digestion.

**Table 7:** Cell viability (log CFU/mL ± SD) within dynamic *in vitro* model and adhesion capability of *B*. *animalis* BF052 from different processes.

Process	Initial count	Gastrointestinal compartment								% Adhesion
		Mouth	Oesophagus-stomach					Duodenum	Ileum	
		2 min	pH 6 10 min	pH 5 10 min	pH 4 10 min	pH 3 30 min	pH 2 30 min	pH 5 30 min	pH 6.5 90 min	
BF052-Caco2	8.13 ± 0.26	-	-	-	-	-	-	-	-	3.19% ± 0.11^A^
BF052-GI test- Caco2	8.47 ± 0.39 ^a^	8.47 ± 0.40 ^a^	8.48 ± 0.42 ^a^	8.46 ± 0.32 ^a^	8.43 ± 0.36 ^a^	8.38 ± 0.41 ^a^	7.21 ± 0.49 ^b^	7.09 ± 0.44 ^b^	6.99 ± 0.49 ^b^	3.81% ± 0.32^A^
BF052-FD- Caco2	8.13 ± 0.18	-	-	-	-	-	-	-	-	3.08% ± 0.15^A^
BF052 -FD-GI test-Caco2	8.34± 0.21 ^a^	8.23 ± 0.21 ^a^	8.32 ± 0.17 ^a^	8.29 ± 0.24 ^a^	8.27 ± 0.21 ^a^	8.19 ± 0.11 ^a^	7.01 ± 0.20 ^b^	6.85 ± 0.11 ^b^	6.77 ± 0.18 ^b^	3.45% ± 0.21^A^
BF052-FD -milk-GI test-Caco2*	8.53 ± 0.21 ^a^	8.49 ± 0.20 ^a^	8.50 ± 0.24 ^a^	8.48 ± 0.23 ^a^	8.43 ± 0.21 ^a^	8.39 ± 0.27 ^a^	7.45 ± 0.14 ^b^	7.35 ± 0.20 ^b^	7.30 ± 0.19 ^b^	3.67% ± 0.50^A^

- The equal superscript lowercase letters in the same row indicate no significant differences between digestion steps (p>0.05)

- The equal superscript capital letter in the last column indicates no significant differences in adhesion percentage for each process (p>0.05)

*In this process, *B*. *animalis* BF052 were freeze-dried (FD) by using 10% skim milk as a cryoprotectant agent and then stored as freeze-dried powders for 1 month at refrigerated temperature following incorporation into a whole pasteurized milk and kept at refrigerated temperature for 2 weeks before exposure through GI (GI) transit followed by adherence assay (Caco2).

In addition, the impact of food manufacturing processes, such as freeze-drying, was also determined and compared with the direct adherence assay. The results showed that no significant differences (P>0.05) in adhesion capability were detected among freeze-dried and non-freeze-dried cells. This result was in contrast to Du Toit et al. [6] who reported that freeze-drying of probiotics was found to have an adverse effect on adhesion capability. Osmotic shock, formation of intracellular ice, and re-crystallization during freeze-drying may damage the biological structures of the cell and probably affect the adhesion ability of probiotics. However, use of an appropriate cryoprotectant during freeze-drying may reduce such adverse changes resulting in the maintenance of the ability of this strain to exhibit probiotic behavior [9]. However, our experiments also demonstrated the effect of freeze-drying process on adhesion ability of probiotics after passage through the conditions of the GI tract. Based on our results, it was observed that the introduction of BF052 through the GI transit may enhance the adhesive ability to Caco-2 cells compared with those of non-challenged conditions. It may be explained that either acid or bile adaptation appeared to affect the *in vitro* adhesion to the intestinal cell line. Also, it was reported that the induction of acid or bile resistance in bifidobacteria may improve cellular surface properties and thus enhance the adhesion ability that favors their potential functionality as probiotics [49,50,51].

Before delivering probiotic-containing products to consumers, probiotic bacteria should survive and retain their functionality not only during storage as freeze-dried cultures but also in the food products into which they are finally formulated [52]. Eventually, it would be beneficial that the strain would be supplemented into the whole pasteurized milk as one of the alternative means for delivering probiotics. In this study, after freeze-drying and the subsequent storage as freeze-dried powder for 1 month, BF052 were sequentially delivered in a whole pasteurized milk as a probiotic vehicle and stored at refrigerated temperatures for two weeks. The survival of the strain throughout the process of simulated digestion was then monitored. Interestingly, the whole production process did not affect the stability of the probiotic properties of BF052, especially the resistance of this strain through the GI transit, including adherence ability. BF052 still displayed a similar ability to withstand GI stresses and exhibited no significant variations (P>0.05) in adhesive ability to Caco-2 cells despite differences in cell preparations. Moreover, it was observed that carriers of probiotic bacteria were involved in affecting the viability and functionality of probiotics during storage and throughout the simulated GI system [53]. Kos et al. [54] studied the effect of whey protein concentrate (WPC) on the viability of *L*. *acidophilus* M92, and found that addition to WPC may protect the cells from the low pH of simulated gastric juice, and even higher concentrations of bile salts. In addition, Madureira et al [48] proposed that whey cheese matrices as a probiotic vehicle were shown to protect *L*. *casei*, *L*. *acidophilus*, and *B*. *animalis* during *in vitro* simulated digestion, compared with their performance in plain MRS medium. Saarela et al. [32] also reported that acid and bile tolerances were better in freeze-dried *B*. *animalis* subsp. lactis E2010 added to pasteurized milk compared with those in phosphate-buffered saline or juice held at 4°C over two weeks. Therefore, several factors may influence the ability of the probiotics to survive in the product and become active when entering the consumer’s GI tract. In this regard, the interactions of probiotics with the food matrix or the starter culture, pH, acidity, temperature, and oxygen content of the product are also important.

Probiotic strains selected for commercial application in foods must retain the characteristics for which they were originally selected [5]. In this report, even though the strains encountered potentially stressful conditions throughout the manufacturing processes and biological barriers during the GI transit, BF052 still maintained its original characteristics. These included the characteristics of survival and tolerance during manufacture and after consumption, and during transit through the stomach and small intestine until adherence to the intestinal epithelium. Therefore, it is anticipated that BF052 retains its probiotic functionality and remains viable at levels necessary to provide health benefits to consumers. However, *in vivo* investigations are still necessary to fully validate its beneficial roles to the health of human hosts.

## Conclusion

It is crucial to investigate interesting strain characteristics in terms of safety and functional aspects for probiotic potential. In this regard, resistance of strains against production, storage, and GI tract stresses is of prime importance. This research demonstrated that *B*. *animalis* BF052 displayed promising probiotic properties and exhibited resilience to adverse conditions not only during industrial processes but also under GI environments before adherence to the intestinal epithelium to exert health-promoting benefits there. Therefore, *B*. *animalis* BF052 studied in this research is a potential probiotic candidate for further development as an effective probiotic starter.

## Supporting Information

S1 Table Data for cell viability (log CFU/ml) in various products after 15 days storage. (DOC)
