# Supplementary material for: Effects of the Food Manufacturing Chain on the Viability and Functionality of Bifidobacterium animalis through Simulated Gastrointestinal Conditions
Source: PLoS One. 2016 Jun 22;11(6):e0157958. doi: 10.1371/journal.pone.0157958 (PMC4917081; doi:10.1371/journal.pone.0157958)
Supplement: S1 Table — (DOC) [file pone.0157958.s001.doc]

**S1 Table: Data for cell viability (log CFU/ml) in various products after 15 days storage**

|  | | **BB12** | | | **BF014** | | | **BF052** | | | **BH053** | | |
| --- | --- | --- | --- | --- | --- | --- | --- | --- | --- | --- | --- | --- | --- |
| **Whole pasteurized milk** | |  | | |  | | |  | | |  | | |
| **Day** | **0** | 7.607 | 7.954 | 7.765 | 8.462 | 8.903 | 8.732 | 8.297 | 8.544 | 8.321 | 8.18 | 7.845 | 7.942 |
|  | **3** | 7.561 | 7.908 | 7.821 | 8.686 | 8.839 | 8.684 | 8.282 | 8.498 | 8.128 | 7.988 | 7.839 | 7.931 |
|  | **6** | 7.533 | 7.813 | 7.742 | 8.431 | 8.651 | 8.458 | 8.265 | 8.556 | 8.552 | 7.757 | 7.898 | 7.816 |
|  | **9** | 7.507 | 7.881 | 7.636 | 8.45 | 8.563 | 8.593 | 8.297 | 8.518 | 8.241 | 7.697 | 7.919 | 7.829 |
|  | **12** | 7.602 | 7.776 | 7.821 | 8.488 | 8.562 | 8.625 | 8.245 | 8.509 | 8.436 | 7.745 | 7.825 | 7.791 |
|  | **15** | 7.620 | 7.707 | 7.684 | 8.342 | 8.633 | 8.571 | 8.217 | 8.491 | 8.408 | 7.735 | 7.819 | 7.770 |
| **Soy milk** | |  | | |  | | |  | | |  | | |
| **Day** | **0** | 8.029 | 7.882 | 7.697 | 8.505 | 8.903 | 8.726 | 8.397 | 8.644 | 8.516 | 8.190 | 8.398 | 8.321 |
|  | **3** | 7.655 | 7.799 | 7.729 | 8.491 | 8.857 | 8.583 | 8.287 | 8.717 | 8.493 | 8.291 | 7.914 | 8.045 |
|  | **6** | 7.653 | 7.707 | 7.714 | 8.42 | 8.716 | 8.547 | 8.193 | 8.684 | 8.547 | 8.021 | 8.431 | 8.162 |
|  | **9** | 7.582 | 7.839 | 7.721 | 8.439 | 8.556 | 8.512 | 8.205 | 8.725 | 8.509 | 7.942 | 8.255 | 8.038 |
|  | **12** | 7.605 | 7.792 | 7.685 | 8.428 | 8.573 | 8.524 | 8.256 | 8.691 | 8.462 | 7.848 | 8.035 | 7.895 |
|  | **15** | 7.611 | 7.740 | 7.662 | 8.394 | 8.602 | 8.497 | 8.201 | 8.568 | 8.385 | 7.787 | 7.973 | 7.914 |
| **Drinking yoghurt** | |  | | |  | | |  | | |  | | |
| **Day** | **0** | 8.019 | 7.882 | 7.936 | 8.505 | 8.691 | 8.672 | 8.397 | 8.644 | 8.518 | 8.190 | 8.368 | 8.275 |
|  | **3** | 7.851 | 7.710 | 7.778 | 8.403 | 8.621 | 8.461 | 8.34 | 8.673 | 8.472 | 8.072 | 8.255 | 8.167 |
|  | **6** | 7.551 | 7.771 | 7.681 | 8.413 | 8.591 | 8.476 | 8.297 | 8.663 | 8.510 | 8.093 | 8.144 | 8.144 |
|  | **9** | 7.565 | 7.748 | 7.662 | 8.538 | 8.653 | 8.512 | 8.398 | 8.568 | 8.473 | 7.887 | 7.932 | 7.897 |
|  | **12** | 7.513 | 7.821 | 7.672 | 8.309 | 8.525 | 8.424 | 8.372 | 8.404 | 8.412 | 7.891 | 7.583 | 7.735 |
|  | **15** | 7.548 | 7.771 | 7.690 | 8.217 | 8.477 | 8.342 | 8.221 | 8.362 | 8.279 | 7.771 | 7.602 | 7.684 |
| **Orange juice** | |  | | |  | | |  | | |  | | |
| **Day** | **0** | 7.963 | 7.782 | 7.864 | 8.505 | 8.791 | 8.649 | 8.297 | 8.344 | 8.320 | 8.190 | 8.285 | 8.268 |
|  | **3** | 7.361 | 7.478 | 7.422 | 8.271 | 8.364 | 8.268 | 8.155 | 8.348 | 8.252 | 7.716 | 7.881 | 7.753 |
|  | **6** | 7.152 | 7.255 | 7.205 | 8.387 | 8.412 | 8.387 | 8.083 | 8.174 | 8.135 | 7.369 | 7.380 | 7.380 |
|  | **9** | 6.991 | 7.079 | 6.991 | 8.267 | 8.327 | 8.302 | 8.004 | 8.072 | 8.072 | 7.182 | 7.255 | 7.217 |
|  | **12** | 6.885 | 6.978 | 6.942 | 8.093 | 7.921 | 8.011 | 7.827 | 7.851 | 7.843 | 7.159 | 7.247 | 7.215 |
|  | **15** | 6.740 | 6.954 | 6.855 | 7.919 | 7.845 | 7.885 | 7.604 | 7.798 | 7.715 | 7.127 | 7.204 | 7.166 |
